# Vertical Skeletal Changes after Extraction and Nonextraction Orthodontic Treatment

**DOI:** 10.1055/s-0042-1749366

**Published:** 2022-07-04

**Authors:** Hafiza Z. Shafique, Rumeesha Zaheer, Abdullah Jan, Alaina T. Mughal, Rooma Shahid, Fareena Ghaffar, Tooba Zahoor, Sundas Mehmood, Ramsha Nawaz, Safia Umar, Mehak Hassan, Muhammad A. Mudasser

**Affiliations:** 1 Orthodontics Department, Armed Forces Institute of Dentistry, Rawalpindi, Pakistan; 2 Orthodontics Department, Dr. Ishrat ul Ibad Khan Institute of Health Sciences, Dow University of Health Sciences, Karachi, Pakistan

**Keywords:** orthodontic space closure, vertical dimensions, cephalometry/methods, artificial intelligence

## Abstract

**Objectives** Due to the constant battle regarding the controversial topic of orthodontic extraction, this study aims to assess the changes in vertical dimensions of patients treated with premolar extractions compared with nonextraction orthodontic patients.

**Materials and Methods** A sample of 60 borderline patients were recruited and divided into extraction and nonextraction groups. Eleven pretreatment cephalometric measurements were recorded using WebCeph and patients were followed-up until the completion of treatment.

**Statistical Analysis** Intragroup and intergroup comparisons were made using paired *t* -test and two-sample independent *t* -test, respectively. The joint significance of differences was measured using F-tests.

**Results** The intragroup comparison revealed that in the extraction group, the vertical dimension was significantly increased posttreatment for four cephalometric measurements, that is, mandibular plane angle ( *p* < 0.05), palatal plane angle ( *p* < 0.05), Frankfort mandibular plane angle ( *p* < 0.05), and y-axis ( *p* < 0.05). In the comparison of the posttreatment values of both groups, the mean differences of the posttreatment values for sella nasion (SN)-gonion (Go)-gnathion (Gn) angle ( *p* = 0.008), the total anterior ( *p* = 0.050), and lower anterior facial heights (AFH; *p* = 0.011) were significantly higher. At the same time, the Jarabak ratio was significantly ( *p* = 0.006) lower in the extraction group than in the nonextraction group.

**Conclusion** The increase in vertical dimension is significantly higher in the extraction group than in the nonextraction group which indicates a significant impact of orthodontic extraction on the vertical dimensions.

## Introduction

Orthodontists and general dental practitioners have spent decades battling orthodontic extractions. Although there are several strategies to manage orthodontic patients, there are two significant schools of thought regarding orthodontic management based on using extraction as a part of treatment plan: 1 2 the supportive and nonsupportive. The supportive school of thought considers orthodontic extractions necessary for management, particularly in patients with a long face. 3 According to this school of thought, one of the primary reasons for choosing extraction as a treatment protocol is its effectiveness in reducing vertical dimension. 1 They also emphasize a lack of solid evidence in the association between TMJ disorders and orthodontic extraction-based treatment plan. 4 5 6 Based on these facts, this ideology promotes orthodontic extractions, particularly in cases with severe crowding or long facial profiles or increased overjet. 7 8

In contrast, the nonsupportive school of thought has a more conservative approach. Their evidence comes from numerous studies that consider premolar extractions a fundamental cause of temporomandibular joint (TMJ) disorders. 4 9 These schools have different theories to explain how these extractions lead to TMJ disorders. Some studies 10 11 report that the molar teeth move anteriorly due to the extraction of premolars, allowing overclosure of the mandible. This effect has been called the “wedge-type effect” in several studies. 1 12 The overclosure of the mandible results in increased stress on the masticatory muscles, causing TMJ pain and other associated problems. Other studies 9 13 report that the maxillary anteriors are overretracted due to premolar extraction resulting in posterior displacement of mandible and condyles which cause TMJ disorders. However, most of the studies 4 5 9 from the nonsupportive group are based on case reports and view-point articles. In addition, a recent systematic review 3 based on 14 studies concluded that extraction treatment does not significantly impact the vertical dimension of patients during orthodontic management.

Although multiple studies have been performed on this controversial topic of orthodontics extraction, no consensus has been established. Our study differs based on strict inclusion criteria. Additionally, to reduce observer error, we incorporated artificial intelligence (AI) software to compare pre- and postcephalometric radiographs. This study aims to assess the changes in the vertical dimension of patients treated with premolar extractions and compare them with the nonextraction group to assess the impact of orthodontic extractions on the vertical dimension of patients.

## Materials and Methods

### Study Design

This prospective cohort study was conducted at Armed Forces Institute of Dentistry (AFID), Pakistan, to evaluate the impact of orthodontic treatment with or without premolar extraction on the skeletal vertical dimension. The study was approved by the Institutional Review Board of AFID (IRB approval no.: 905/Trg-ABP1k2) and was conducted from April 2019 to August 2021.

### Sample Inclusion and Exclusion Criteria

The sample included 36.7% males ( *n* = 22) and 63.3% females ( *n* = 38). The mean age of our sample was 16.63 ± 2.7 years. The inclusion criteria for the study had both male and female patients having a complete set of permanent teeth except third molars. An A point, Nasion, B point (ANB) of −1 to 6 degrees, angle's class I or II malocclusion, crowding 5 to 9 mm, and overjet of 4 to 6 mm were also part of the inclusion criteria. Any patient with dentofacial deformity, transverse discrepancy, temporomandibular joint disorders (TMDs), or any previous history of orthodontic/orthognathic treatment was excluded from the study. Patients with impacted, missing, or decayed teeth were also excluded.

Out of the 60 borderline cases selected based on the abovementioned inclusion criteria, 30 patients were selected in extraction and nonextraction groups. Informed consent was obtained from all the selected individuals after explaining the nature and purpose of the study, relevant radiographic examinations, and history.

### Radiographic Analysis

The pre and posttreatment cephalograms were taken for all patients, and cephalometric measurements were recorded after cephalometric tracing using WebCeph software. 14 WebCeph was designed and coded by an Orthodontist. It was manufactured on November 10, 2020. It is an online orthodontic and orthognathic platform for dental clinicians to upload their cephalograms, and the software uses AI to identify critical anatomical landmarks and completes cephalometric tracing in seconds. WebCeph also allows manual marking and editing of the anatomical landmarks followed by automatic calculation of measurements. 14 15 This automated cephalometric tracing saves time and effort and reduces the chances of observer error. Furthermore, it avoids errors due to poor calibration and low interexaminer agreement scores. Several studies support the use of AI-based software like WebCeph to identify multiple cephalometric landmarks and analysis. 15 16 17

All the radiographs in the study were taken on two radiographic units, Sirona Dental Systems GmbH and Carestream Health, Inc., at the AFID. The Sirona Dental Systems GmbH is manufactured by Sirona Dental Systems Inc, which is a U.S. based company, while Carestream Health is a worldwide imaging company with its headquarters in Rochester, New York, United States.

Two trained examiners recorded the lateral cephalograms of study participants following the standard protocol. The patients were positioned in the cephalostat with the Frankfort plane parallel to the floor, the sagittal plane perpendicular to the path of the X-rays, neutral head position with unstrained lips and teeth in centric occlusion. The image size calibration was also done with WebCeph. Finally, after identifying landmarks and tracing cephalograms, 11 commonly used variables that measure the skeletal vertical dimension were employed to assess and compare the impact of the two different treatment approaches (extraction vs. nonextraction). Table 1 defines these 11 variables, 3 of which were linear measurements (in mm), 1 was a ratio of linear measurements, and the remaining 7 were angular (in degrees).

**Table 1:** Definition of cephalometric measurements for analyses 8 10 16 17 18

Cephalometric measurements recorded for analyses	
Anterior facial height (AFH)	The distance between nasion (N) and menton (Me) 26
Posterior facial height (PFH)	The distance between sella (S) and gonion (Go) 26
Lower anterior facial height (LAFH)	The distance between anterior nasal spine (ANS) and Me 26
Jarabak's ratio (PFH/AFH)	Ratio of the distance between S and Go to the distance between N and Me, 26 i.e., the relation of posterior to anterior facial height (SGo:Me) 20
BJORK sum	The sum of posterior angles, i.e., saddle + articulare + gonial = sum of the N–S–Ar, S–Ar–Go, and Ar–Go–Me angles 27
Gonial angle	The angle at the intersection of the lines tangent to the posterior border of the ramus and the inferior border of the mandible 27
SN-Go-Me	The angle between sella nasion (SN) plane and mandibular plane (MP) 10
MP angle (MPA) in McNamara's analysis	The angle formed by the intersection of Me–Go and orbital–porion lines 26
Frankfort MP angle (FMA)	Frankfurt horizontal (FH) plane to MP derived by the line connecting the landmarks Go and Me 12
Palatal plane angle (PPA)	The angle between FH plane and palatal plane (PP)
Y-axis (Down's analysis)	Y-axis is the angle formed at the intersection of the line S to gnathion to FH plane. 12

**Fig. 1:**
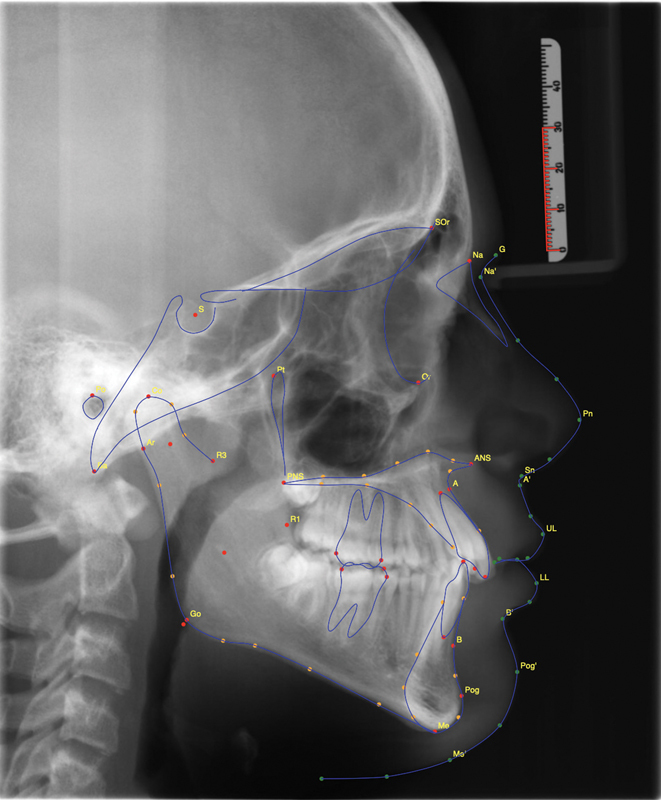
Image taken from WebCeph and landmarks pointed.

The cephalometric points, lines, and measurements used in this study to evaluate the vertical dimension of patients' pre- and postorthodontic treatment are shown in Fig. 1 .

### Statistical Analysis

The sample size calculations were done using PS software version 3.1.6, assuming that the difference in the response of matched pairs (pre- and posttreatment) is usually distributed with a standard deviation (SD) of 2.75 degrees. If the true difference in the mean response of matched pairs is 1.7 degrees, we will need to study 30 pairs of patients to be able to reject the null hypothesis setting the significance level at 0.05 with a probability (power) of 0.9.

The normal distribution of the sample was assessed using QQ plots for various parameters of vertical dimension. The data were normally distributed, and, therefore, Student's *t* -tests were used to assess the differences between the extraction and nonextraction groups' pre- and posttreatment cephalometric measurements based on the various parameters of vertical dimension.

For intragroup comparison, the mean and SD of pretreatment values and the posttreatment values of the 11 cephalometric parameters were recorded for each group (extraction and nonextraction). The difference between these pre- and posttreatment values was calculated and, then, using a paired *t* -test, these values were compared to see if the differences were statistically significant.

For the intergroup comparison, the pretreatment values were deducted from the posttreatment values for each of the 11 cephalometric variables to assess the alterations produced by orthodontic treatment. The changes in these cephalometric variables resulting from orthodontic treatment were compared between two groups (extraction vs. nonextraction) for statistical differences using a two-sample independent *t* -test. The joint significance of differences was measured using F-tests. All analyses were performed by the computer program SPSS, version 26.

## Results

The final study sample ( *n* = 60) had 30 patients in each extraction group and nonextraction group. The overall sample comprised of 36.7% males ( *n* = 22) and 63.3% females ( *n* = 38). The extraction group had a similar distribution with 33% males and 67% females, whereas the nonextraction group had slightly more males (40%). The overall mean age of participants was 16.63 ± 2.72 years, with the majority (60%, *n* = 36) within the age range of 15 to 18 years. About 82% of the sample belonged to a high socioeconomic status (SES).

The study participants were divided into three categories based on the ANB values regarding the skeletal jaw relationship. Based on the ANB values, 65% ( *n* = 39) of the sample belonged to skeletal class I, 30% to class II, and only 5% to class III. About 63% ( *n* = 19) of the participants from the extraction group and 67% ( *n* = 20) from the nonextraction groups had a skeletal class-I relationship. About 70% of patients ( *n* = 42) had a dental class-I occlusal relationship, whereas 30% had class II. Of those from the extraction group, 73% ( *n* = 22) had a dental class-I relationship, while approximately 67% ( *n* = 20) from the nonextraction group had a dental class-I relationship ( Table 2 ).

**Table 2:** Intragroup comparisons of cephalometric values pre- and postorthodontic treatments

Variables	Operational definition	Extraction group		Nonextraction group		Total	
		***n***	**%**	***n***	**%**	***n***	**%**
Skeletal relationship	ANB 2 − 4	19	63.3	20	66.7	39	65
	ANB 5 − 6	10	33.3	8	26.7	18	30
	ANB −1 − +1	1	3.3	2	6.7	3	5
	Total	30	100	30	100	60	100
Dental class	Class I	22	73.3	20	66.7	42	70
	Class II	8	26.7	10	33.3	18	30
	Total	30	100	30	100	60	100

### IntraGroup Comparison Results

The intragroup comparison in the extraction group revealed that 4 out of 11 variables were statistically significant at *p* < 0.05. These variables included mandibular plane angle (MPA; *p* = 0.012), palatal plane angle (PPA; *p* = 0.009), Frankfort mandibular-plane angle (FMA; *p* = 0.011), and y-axis ( *p* = 0.015). These values show a significant impact of orthodontic extraction on the vertical dimension of patients and reject the null hypothesis. The negative values for the mean differences (pretreatment − posttreatment) show a rise in the vertical dimensions postorthodontic treatment. However, in the nonextraction group, none of the 11 variables had a significant mean difference at a *p* -value of <0.05. The negative values of the mean difference indicated an increase in the vertical dimension but these differences were not statistically significant ( Table 3 ).

**Table 3:** Intragroup comparisons of cephalometric values pre- and postorthodontic treatments

Variables	Extraction group				Nonextraction group			
	Pretreatment Mean (SD)	Posttreatment Mean (SD)	Mean difference (pre − post)	*p* -Value	Pretreatment Mean (SD)	Posttreatment Mean (SD)	Mean difference (pre − post)	*p* -Value
AFH	120.6 (12.9)	122.9 (11.7)	−2.312	0.140	116.3 (10.4)	117.4 (9.7)	−1.043	0.508
PFH	78.1 (9.5)	79.6 (7.4)	−1.503	0.210	76.9 (11.9)	80.0 (6.2)	−3.144	0.168
LAFH	69.3 (7.2)	72.2 (7.2)	−2.864	0.023	67.0 (7.1)	65.8 (11.2)	1.214	0.441
Jarabak's ratio	64.8 (5.1)	64.9 (4.5)	−0.092	0.835	70.3 (10.4)	68.3 (4.7)	1.967	0.220
Bjork sum	393.9 (6.4)	393.7 (8.7)	0.158	0.904	389.9 (6.5)	390.3 (5.9)	−0.343	0.452
Gonial	121.7 (6.6)	122.0 (6.4)	−0.288	0.748	119.2 (7.1)	119.1 (6.8)	0.127	0.834
SN-Go-Gn	34.2 (6.1)	34.6 (6.1)	−0.440	0.387	32.6 (16.0)	30.3 (5.9)	2.290	0.426
MPA	24.0 (6.0)	25.3 (5.8)	−1.23	0.012	24.1 (9.7)	24.0 (8.8)	0.059	0.978
FMA	24.0 (6.0)	25.3 (5.8)	−1.274	0.011	22.2 (5.6)	22.6 (5.5)	−.462	0.243
PPA	−1.5 (3.0)	−0.3 (2.4)	−1.189	0.009	0.6 (2.4)	0.6 (2.4)	0.075	0.798
Y-axis	59.4 (3.3)	60.3 (3.1)	−0.939	0.015	58.7 (6.6)	59.2 (3.9)	−0.503	0.563

Abbreviations: AFA, anterior facial height; FMA, Frankfort mandibular-plane angle; Gn, gnathion; Go, gonion; LAFH, lower anterior facial height; MPA, mandibular plane angle; PFA, posterior facial height; post, posttreatment; PPA, palatal plane angle; pre, pretreatment; SD, standard deviation.

### InterGroup Comparison Results

In comparing the posttreatment values of the extraction group with the nonextraction group, the mean differences of the posttreatment values for 4 out of 11 cephalometric variables were statistically significant, whereas 7 were not. The four cephalometric variables with significant results included sella nasion (SN)-gonion (Go)-gnathion (Gn) angle ( *p* = 0.008), the anterior ( *p* = 0.050) and lower anterior facial heights (LAFH; *p* = 0.011), and the Jarabak ratio ( *p* = 0.006). The mean difference between the two groups for these four variables was 4.31, 5.54, 6.42, and −3.42, respectively ( Table 4 ).

**Table 4:** Intergroup comparisons of posttreatment cephalometric values for extraction versus nonextraction groups

Variable for cephalometric measurement	F a	*t*	Significance (two-tailed)	Mean difference	Standard error difference	95% confidence interval	
						Lower	Upper
Post Bjork (degree)	0.872	1.770	0.082	3.412	1.928	−0.447	7.271
Post FMA (degree)	0.017	1.826	0.073	2.660	1.457	−0.256	5.576
Post-gonial (degree)	0.185	1.718	0.091	2.937	1.710	−0.485	6.360
Post-SN-Go-Gn (degree)	0.080	2.771	0.008	4.308	1.555	1.196	7.421
Post-PFH (mm)	0.095	−0.208	0.836	−0.368	1.767	−3.906	3.170
Post-AFH (mm)	0.047	2.001	0.050	5.539	2.769	−0.003	11.082
Post-Jarabak's ratio	0.024	−2.872	0.006	−3.416	1.189	−5.797	−1.035
Post-LAFH (mm)	1.086	2.639	0.011	6.419	2.433	1.549	11.288
Post-MPA (degree)	0.823	0.657	0.514	1.264	1.922	−2.585	5.112
Post-y-axis (ͦ(degree)	1.425	1.191	0.238	1.095	0.919	−0.745	2.935
Post-PPA (degree)	0.026	−1.330	0.189	-0.825	0.620	−2.066	0.416

Abbreviations: AFA, anterior facial height; FMA, Frankfort mandibular-plane angle; Gn, gnathion; Go, gonion; LAFH, lower anterior facial height; MPA, mandibular plane angle; N, nasion; PFA, posterior facial height; PPA, palatal plane angle. S, sella.

a based on the equal assumption of variance, calculated using Levene's test for equality of variance.

## Discussion

The present study aimed to assess the impact of orthodontic extraction on the vertical dimensions of a sample of borderline cases with ANB ranging between −1 and 6 degrees and dental malocclusion of class I or II. These cases could either be treated with a treatment plan involving extractions of two or all four premolars or could be treated with a nonextraction treatment plan based on the clinician's decision. The study not only compared the pre- and posttreatment cephalometric measures of vertical change in the orthodontic extraction group but also assessed change in the nonextraction group. In addition, the study compared the changes in the vertical facial dimension of the extraction group with the nonextraction group. These analyses were conducted after following 60 patients (30 in each group) until the completion of their treatment.

The intragroup comparison results had negative mean difference values (pretreatment values – posttreatment values), indicating a rise in the vertical proportions after the completion of treatment. Out of the 11 cephalometric parameters evaluated for vertical change, 4 were significantly higher in the extraction group posttreatment, whereas none of them was significantly high in the nonextraction group at *p* < 0.05. The variables that showed a significant increase in the extraction group included MPA ( *p* = 0.012), PPA ( *p* = 0.009), FMA ( *p* = 0.011), and y-axis ( *p* = 0.015). Six more variables (posterior facial height (PFH), AFH, LAFH, Jarabak's ratio, gonial angle, and SN-GoGn angle) showed an increase in vertical dimension posttreatment but the results were not statistically significant. These results were in agreement with Staggers 18 that did not find a significant increase in the LAFH and Jarabak's ratio. Kim et al 10 reported a significant increase in the AFH, and a study by Upadhyay et al 8 also reported a significant increase in PFH and Jarabak's ratio in orthodontic extraction cases. This increase in vertical dimensions of patients treated with an extraction plan can be explained by growth since most of the patients were of growing age (11–15 years) and because the extrusion was uncontrolled. Due to the extraction of premolars, the molars moved anteriorly. Despite this forward movement, extrusion of the molars was seen because of interarch mechanics. This extrusion leads to downward and backward rotation of the mandible, increasing the vertical facial dimension, thus emphasizing the importance that needs to be given to the extrusive mechanics of orthodontics. Thus, particular caution is critical in the treatment planning of hyperdivergent patients. 19

On the other hand, Kirschneck et al 20 and Kim et al 21 reported a decrease in the vertical dimension based on the gonial angle. Few studies 11 22 reported no influence on the vertical dimension of orthodontic extraction cases. A recent systematic review 20 also concluded that the extraction of all four premolars did not affect the skeletal vertical dimension of orthodontic patients.

In the nonextraction group, the mean difference of cephalometric measures showed mixed results but was insignificant at *p* < 0.05. Some variables showed an increase in the vertical dimension (i.e., AFH, PFH, Bjork, FMA, and y-axis), while others decreased (i.e., LAFH, Jarabak's ratio, gonial angle, SN-Go-Gn angle, MPA, and PPA). These results were similar to Beit et al 12 who reported an increase in some variables and a decrease in others. Similar to our findings, their study reported an increase in the y-axis and a decrease in the gonial angle. Contrary to our findings, Kirschneck et al 20 reported a decrease in AFH in the nonextraction group.

Regarding the intergroup comparison of posttreatment cephalometric values, the extraction group showed significantly high vertical dimensions for four variables compared with the nonextraction group ( Table 4 ). These variables included SN-Go-Gn angle ( *p* = 0.008), the anterior ( *p* = 0.050), and LAFHs ( *p* = 0.011), and Jarabak's ratio ( *p* = 0.006). The mean difference in the posttreatment values of these four variables between the two groups were 4.31, 5.54, 6.42, and −3.42, respectively. Even though most of the studies 1 18 23 have shown an insignificant difference in the two groups, our results showed a significant increase based on four variables and an insignificant increase based on five variables (Bjork sum, FMA, gonial angle, MPA, and y-axis). Beit et al 12 also stated statistically insignificant differences in the mean values for five cephalometric variables and significant differences for two variables (i.e., mandibular plane to the cranial base and the y-axis) between the two groups.

## Strengths and Limitations

The study had several strengths, such as prospective study design, adequate sample size, and uniform distribution of the sample into two groups based on age, gender, SES, and treatment plan (extraction vs. nonextraction). We used a software that was based on AI to automatically locate landmarks and carry out analysis. This reduced observer error and was cost and time effective. AI-based cephalometric analysis is rapidly gaining popularity among orthodontists and is gaining precedence over manual tracing. It also enables accurate diagnosis and treatment planning. 24 25 The main limitation of this study was the extrusive mechanics of orthodontic treatment. In addition, the two-dimensional radiographic analysis using lateral cephalogram has limited accuracy and is prone to errors and superimposition, despite being the most popular diagnostic aid used in orthodontics clinics for treatment planning. Furthermore, the radiographic measurements were calculated using WebCeph, an online software that uses AI. The software is also prone to errors and can bias the results. Lastly, similar to many other studies, our study did not follow-up enough to evaluate relapse.

## Conclusion

Our study found only 4 out of 11 variables significantly high in postorthodontic treatment involving extractions. However, no variable was significant in cases that were treated without extraction. Four variables were significantly different when comparing posttreatment values of extraction versus nonextraction groups. Therefore, based on our study's findings, it seems reasonable to conclude that the increase in vertical is due to a lack of attention given to orthodontic mechanics. If caution is taken to control the extrusion of molars after premolar extraction, there will most likely be no significant change in the vertical dimension of orthodontic patients. It is also recommended to be more cautious in patients with hyperdivergent facial profiles.
